# Efficacy of prophylactic laxatives against opioid-induced constipation: retrospective propensity score matching analysis

**DOI:** 10.1007/s00520-025-09154-w

**Published:** 2025-01-21

**Authors:** Yuka Okuda, Toshiyuki Kuriyama, Yoshi Tsukiyama, Toshio Shimokawa, Ke Wan, Tomoyuki Kawamata

**Affiliations:** 1 Department of Anesthesiology, School of Medicine, Wakayama Medical University, 811-1 Kimiidera, Wakayama-Shi, 641-8509 Japan; 2 Clinical Study Support Center, Wakayama Medical University, Wakayama, Japan

**Keywords:** Opioid-induced constipation, Cancer pain, Prophylactic laxatives, ECOG PS

## Abstract

**Purpose:**

Opioid-induced constipation (OIC) is problematic for patients with cancer receiving opioid therapy. Some guidelines recommend initiating regular laxatives at the same time as opioid analgesics. However, the effectiveness of prophylactic laxatives on OIC has not been widely demonstrated. We therefore examined the incidence of OIC among patients with and without prophylactic laxatives within one week after initiating strong opioid analgesics and the factors associated with the incidence of OIC.

**Methods:**

Eligible patients were adults with cancer for whom strong opioid analgesics were initiated after admission and who remained hospitalized for over a week. Propensity score matching analysis was used to compare outcomes after adjusting for patient background.

**Results:**

In total, 928 patients were enrolled, from which 536 were selected after propensity score matching analysis. The incidence of OIC in patients with prophylactic laxatives was not significantly different from that without prophylactic laxatives (48.1% vs 48.9%, odds ratio (OR) = 0.97, 95% confidence interval (CI): 0.69–1.36). In multivariable logistic analysis, age ≥ 65 years (OR = 1.53, 95% CI: 1.07–2.19) and Eastern Cooperative Oncology Group Performance Status (ECOG PS) ≥ 3 (OR = 1.69, 95% CI: 1.04–2.77) were associated with a higher incidence of OIC.

**Conclusion:**

Prophylactic laxatives did not affect the incidence of OIC in our study. Our results suggest that prophylactic laxatives are not necessarily required when initiating opioid therapy. In addition, we found that age ≥ 65 years and ECOG PS ≥ 3 were associated in our study with a higher incidence of OIC.

**Supplementary Information:**

The online version contains supplementary material available at 10.1007/s00520-025-09154-w.

## Introduction

Opioid analgesics are widely used for moderate to severe pain in patients with cancer [1]. However, constipation is a common side effect of opioid analgesics. Among opioid receptors, μ opioid receptors are related to opioid-induced constipation (OIC). They are mainly found in the stomach and proximal colon, on the membranes of intestinal muscle cells, myenteric and submucosal neurons [2, 3]. The activation of these μ opioid receptors reportedly inhibits intestinal movement and intestinal mucosal secretion, promotes intestinal fluid absorption, and increases anal sphincter contraction [4]. Constipation reportedly occurs in 33–55% in patients with cancer within one week after they initiate strong opioid analgesics [5]. In addition, OIC could result in avoidance of opioid use, leading to inadequate pain relief, biopsychosocial burden, impaired quality of life (QOL), and subsequently a greater financial burden [3, 6–10]. A previous study has shown that healthcare providers often do not warn patients about constipation, a common side effect of opioid use, which has led to an increase in the number of patients complaining of constipation [10]. Therefore, we suggest that OIC requires attention.

The use of traditional laxatives (osmotic laxatives and stimulant laxatives) is recommended as first-line therapy [11, 12]. In addition, peripherally-acting μ opioid receptor antagonist (PAMORA) including naldemedine is recommended for patients with OIC refractory to traditional laxatives [11, 12]. The European Association for Palliative Care [13] and the National Institute of Clinical Evidence [14] recommend prescribing prophylactic laxatives to prevent OIC. A clinical guideline of the Japanese Society of Palliative Medicine also recommends initiating regular laxatives at the same time as opioid analgesics; this is based on daily clinical experience but not clinical evidence [12]. Hamano et al. recently demonstrated a preventive effect of naldemedine on constipation and improvement in constipation-related QOL in patients with cancer in whom regular administration of strong opioid analgesics was initiated [15]. However, naldemedine is not currently approved for prophylactic use in OIC. Thus, the effectiveness of clinically available prophylactic laxatives for OIC has not been sufficiently demonstrated. To our knowledge, two studies have examined the preventive effects on OIC [5, 16]. First, the OIC-J study prospectively examined the incidence of OIC with or without prophylactic laxatives as a secondary endpoint [16]. The cumulative incidence of OIC according to the Rome IV diagnostic criteria was 48% in patients who received prophylactic laxatives (constipation agents initiated at the same time of opioid therapy) and 65% of patients who received no prophylactic laxatives. However, the incidence of OIC in patients with prophylactic laxatives was not statistically evaluated in comparison with that of patients without prophylactic laxatives. Therefore, the OIC-J study did not clarify the effectiveness of prophylactic laxatives for OIC. Second, the J-RIGID study retrospectively examined the effectiveness of prophylactic laxatives using the different criteria for OIC from those used in the OIC-J study [5]. Prophylactic laxatives administered within 7 days after initiating opioid analgesics statistically significantly decreased the incidence of OIC [5], but there was no adjustment for confounding bias in patient background.

We examined the incidence of OIC as a primary endpoint and made statistical comparisons for the first time. Although retrospective in nature, we used propensity score matching analysis to adjust for comparative bias in patient background as much as possible to enhance the level of evidence. We also investigated risk factors for OIC.

## Materials and methods

### Study design and patients

Our study was approved by the Wakayama Medical University Certified Review Board (CRB5180004) and was conducted in compliance with the Declaration of Helsinki and Ethical Guidelines for Medical and Health Research Involving Human Subjects. We conducted a retrospective, single-institution observational cohort study to assess the clinical effects of prophylactic laxatives on OIC. All clinical data were obtained from electronic medical records. Eligible patients were adult (≥ 20 years) cancer inpatients who initiated regular administration of strong opioid analgesics after admission to our hospital and who remained hospitalized for over a week between January 2012 and January 2023. We excluded patients with a history of colostomy and those missing description-based data on defecation. Owing to the anonymous nature of the data, informed consent was waived and a disclosure document was posted.

### Assessments

We diagnosed OIC when there was a stool-free interval of ≥ 72-h within one week after initiating strong opioid analgesics, in accordance with a previous study [5]. We defined prophylactic laxatives as regularly administered laxatives initiated on the same day (day 0) as opioid therapy according to the OIC-J study. Additional laxatives were initiated as needed between the next day (day 1) and seven days (day 7) after initiating opioid therapy. In patients without prophylactic laxatives, laxatives were initiated as needed between day 1 and day 7 after initiating opioid therapy, but the distinction lies in there being no initial administration on the same day like in the prophylactic laxatives.

The primary endpoint of this study was the incidence of OIC in patients with and without prophylactic laxatives. In addition, we examined factors associated with the incidence of OIC. Explanatory variables were selected based on previous studies [17–20]. Patient clinical demographics included age (≥ 65 years), sex, body mass index (BMI) (≥ 21 kg/m2), type of cancer (gastrointestinal or not), Eastern Cooperative Oncology Group Performance Status (ECOG PS) ≥ 3, presence of cancerous peritonitis, previous gynecological or abdominal surgery, presence of diabetes, use of benzodiazepine, timing of anti-cancer treatment, use of weak opioid analgesics before initiating strong opioid analgesics, types of laxatives used, use of laxatives before initiating strong opioid analgesics and their types, and the administration route and morphine equivalent daily dose of opioid analgesics.

We also examined the effect of the types of laxatives on the incidence of OIC. Laxatives were classified into four types: osmotic laxatives, stimulant laxatives, PAMORA, and others [21]. ‘Osmotic laxatives’ included magnesium oxide, lactulose and polyethylene glycol, ‘stimulant laxatives’ included sennoside and picosulfate, PAMORA was naldemedine, and then ‘others’ comprised linaclotide, lubiprostone, and elobixibat. In addition, ‘combination therapy’ was the use of more than 2 types.

### Statistical analysis

Categorical variables, summarized as frequency and proportion, were compared using the Fisher's exact test. Continuous variables, summarized as median (range), were compared using the Wilcoxon test.

We used propensity score matching analysis to adjust for comparative bias in patient background as much as possible to enhance the level of evidence. A propensity score was calculated using the multivariate logistic regression with following covariates: age, sex, BMI, type of cancer, ECOG PS, presence of cancerous peritonitis, previous gynecological or abdominal surgery, presence of diabetes, use of benzodiazepine, timing of cancer treatment, use of weak opioid analgesics, use of laxatives before initiating strong opioid analgesics, and the types of opioid analgesics and their administration route. We performed propensity score matching analysis between patients with and without prophylactic laxatives. We used the caliper width of 0.20 for one-to-one matching analysis and the nearest neighbor matching on the logit of the propensity score. The propensity score-matched data were analyzed for the incidence of OIC in patients with and without prophylactic laxatives using the Fisher’s exact test. Multivariable logistic analysis was performed to clarify factors associated with the incidence of OIC. To compare patient background, the chi-squared test was used for categorical variables and the Wilcoxon rank sum test for continuous variables.

In a clinical setting, laxatives are often initiated within 3 days after starting opioid therapy. Therefore, we redefined prophylactic laxatives as those initiated regularly on day 0, 1, or 2 after starting strong opioid analgesics. We performed propensity score matching analysis again, and we then performed sensitivity analysis to compare the incidence of OIC in patients with and without redefined prophylactic laxatives. All analyses were carried out using JMP® version 14.1.0 (SAS Institute, Cary, NC, USA). All statistical tests were performed with a two‐sided significance level of 0.05.

## Results

### Characteristics of all eligible patients

**Fig. 1:**
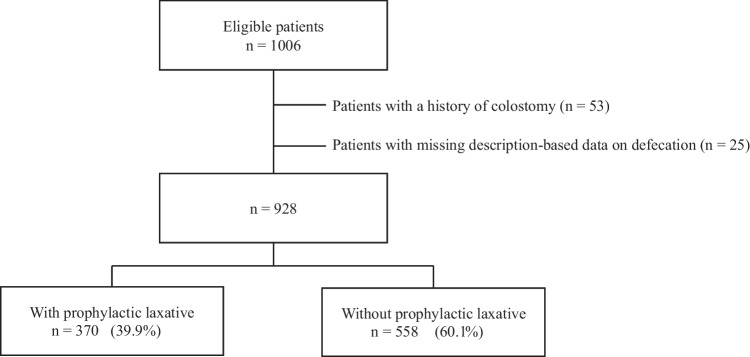
Patient flow diagram

Between January 2012 and January 2023, 1006 patients initiated regular administration of strong opioid analgesics for cancer pain after admission to our hospital and were hospitalized for over a week. We excluded 78 patients: 53 due to a history of colostomy, and 25 because they lacked description about defecation due to missing description-based data on defecation in electronic medical records. Accordingly, 928 patients met the eligibility criteria of our study, 370 (39.9%) of whom received prophylactic laxatives (Fig. 1). Characteristics of all eligible patients are shown in Table 1. There were significant differences between patients with and without prophylactic laxatives in sex, BMI, type of cancer, ECOG PS, timing of anti-cancer treatment, types and administration route of opioid analgesics, and use of laxatives before initiating strong opioid analgesics.

**Table 1:** Characteristics of all eligible patients

		All (*n* = 928)	With prophylactic laxatives (*n* = 370)	Without prophylactic laxatives (*n* = 558)	*P* value
Age (years)	Median (range) ≥65 (%) <65 (%)	67 (22–93) 530 (57.1) 398 (42.9)	68 (29–92) 211 (57.0) 159 (43.0)	67 (22–93) 319 (57.2) 239 (42.8)	0.636
					0.967
Sex	Male (%) Female (%)	600 (64.7) 328 (35.3)	257 (69.5) 113 (30.5)	343 (61.5) 215 (38.5)	0.014*
BMI (kg/m2)	Median (range) ≥21 (%) <21 (%)	21 (11.7–37.1) 472 (50.9) 456 (49.1)	21.4 (13.1–37.1) 211 (57.0) 159 (43.0)	20.7 (11.7–36.6) 261 (46.8) 297 (53.2)	0.002*
					0.001*
Type of cancer	Gastrointestinal (%) Others (%)	87 (9.4) 841 (90.6)	25 (6.8) 345 (93.2)	62 (11.1) 496 (88.9)	0.023*
ECOG PS	≤2 (%) ≥3 (%)	623 (67.1) 305 (32.9)	271 (73.2) 99 (26.8)	352 (63.1) 206 (36.9)	0.001*
Timing of anti-cancer treatment	Anticancer treatment (%) Best Supportive care (%)	619 (66.7) 309 (33.3)	271 (73.2) 99 (26.8)	348 (62.4) 210 (37.6)	<.001*
Presence of cancerous peritonitis	Yes (%) No (%)	174 (18.8) 754 (81.2)	61 (16.5) 309 (83.5)	113 (20.3) 445 (79.7)	0.148
Previous gynecological or abdominal surgery	Yes (%) No (%)	263 (28.3) 665 (71.7)	166 (44.9) 204 (55.1)	97 (17.3) 461 (82.6)	0.317
Presence of diabetes	Yes (%) No (%)	163 (17.6) 765 (82.4)	68 (18.4) 302 (81.6)	95 (17.0) 463 (83.0)	0.597
Use of benzodiazepine	Yes (%) No (%)	246 (26.5) 682 (73.5)	88 (23.8) 282 (76.2)	158 (28.3) 400 (71.7)	0.124
Types of opioid analgesics	Morphine (%) Oxycodone (%) Fentanyl (%) Hydromorphone (%) Tapentadol (%)	56 (6.0) 629 (67.8) 212 (22.8) 74 (8.0) 25 (2.7)	20 (5.4) 278 (75.1) 39 (10.5) 44 (11.9) 16 (4.3)	36 (6.5) 351 (62.9) 173 (31.0) 30 (5.4) 9 (1.6)	0.510 <.001* <.001* <.001* 0.014*
Administration route of opioid analgesics	Oral (%) Not oral (%)	645 (69.5) 283 (30.5)	322 (87.0) 48 (13.0)	323 (57.9) 235 (42.1)	<.001*
Daily dose of opioid analgesics (morphine equivalent) (mg/day)	Median (range)	30 (9.6–210)	30 (9.6–120)	30 (9.6–210)	0.051
Use of weak opioid analgesics	Yes (%) No (%)	355 (38.3) 573 (61.7)	138 (37.3) 232 (62.7)	217 (38.9) 341 (61.1)	0.625
Use of laxatives before initiating strong opioid analgesics	Yes (%) No (%)	335 (36.1) 593 (63.9)	90 (24.3) 280 (75.7)	245 (43.9) 313 (56.1)	<.001*
Types of prophylactic laxatives	Osmotic laxatives (%) Stimulant Laxatives (%) PAMORA (%) Others (%) Combination therapy (%)	194 (20.9) 53 (5.7) 85 (9.2) 11 (1.2) 27 (2.9)	194 (52.4) 53 (14.3) 85 (23.0) 11 (3.0) 27 (7.3)	NA	NA

*BMI* body mass index, *ECOG PS*, eastern cooperative oncology group performance status, *BSC* best supportive care, *PAMORA* peripherally-acting μ opioid receptor antagonist, *NA* not applicable

*P < 0.05

### Characteristics of after propensity score matching analysis

After propensity score matching analysis, 536 patients were selected. The patients' characteristics were similar between patients with and without prophylactic laxatives. There were no significant differences between the two groups (Supplementary Table 1).

Among patients without prophylactic laxatives, 104 of 268 (38.8%) required laxatives within one week of initiating opioid therapy. The median time from initiating opioid therapy to administering laxatives was 3 days (range: 1–7 days). The remaining 164 patients did not receive any laxatives. Among the patients with prophylactic laxatives, 114 of 268 patients had received additional laxatives, and the median time from initiating opioid therapy to administering additional laxatives was also 3 days (range: 1–7 days). The remaining 154 patients never received any additional laxatives.

### Primary endpoint

The incidence of OIC in patients with and without prophylactic laxatives is shown in Table 2. Before propensity score matching analysis, there was no statistically significant difference in the incidence of OIC between patients with and without prophylactic laxatives (47.8% vs 49.8%, odds ratio (OR) = 0.92, 95% confidence interval (CI): 0.71–1.21) (Table 2). After propensity score matching analysis, the incidence of OIC in patients with prophylactic laxatives was not significantly different from that in patients without prophylactic laxatives (48.1% vs 48.9%, OR = 0.97, 95% CI: 0.69–1.36) (Table 3).

**Table 2:** Incidence of OIC in patients with and without prophylactic laxatives. Before propensity score matching analysis

		Prophylactic laxatives		OR (95% CI)	*P* value
		Yes (*n* = 370)	No (*n* = 558)		
OIC	Yes (*n* = 455)	177	278	0.92 (0.71–1.21)	0.554
	No (*n* = 473)	193	280

**Table 3:** Incidence of OIC in patients with and without prophylactic laxatives. After propensity score matching analysis

		Prophylactic laxatives		OR (95% CI)	*P* value
		Yes (*n* = 268)	No (*n* = 268)		
OIC	Yes (*n* = 260)	129	131	0.97 (0.69–1.36)	0.863
	No (*n* = 276)	139	137		

The types of prophylactic laxatives used are shown in Supplementary Table 1. The incidence of OIC for osmotic laxatives, stimulant laxatives, PAMORA, others, and their combinations were 54.9%, 43.6%, 36.4%, 57.1%, and 47.8%, respectively. The p-value in the Fisher's exact test was 0.148, and there was no significant difference in the incidence of OIC according to the types of prophylactic laxatives used.

### Factors associated with the incidence of OIC

**Fig. 2:**
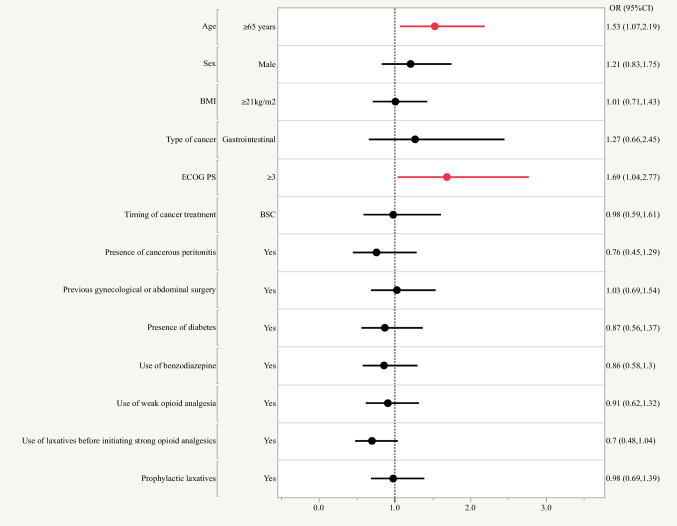
Factors associated with the incidence of OIC after propensity score matching analysis using multivariable logistic analysis. Age ≥ 65 years and ECOG PS ≥ 3 were associated with a higher incidence of OIC. BMI, body mass index; ECOG PS, Eastern Cooperative Oncology Group Performance Status; BSC, Best Supportive Care

Next, we examined the factors associated with the incidence of OIC after propensity score matching analysis (Fig. 2). Multivariable logistic analysis showed that age ≥ 65 years (OR = 1.53, 95% CI: 1.07–2.19) and ECOG PS ≥ 3 (OR = 1.69, 95% CI: 1.04–2.77) were associated with a higher incidence of OIC.

### Sensitivity analysis

For sensitivity analysis, prophylactic laxatives were redefined as prophylactic laxatives as initiated regularly on day 0, 1, or 2, and 446 patients (48.1%) received the redefined prophylactic laxatives. Then propensity score matching analysis was performed and 289 patients in each group were selected for sensitivity analysis. Before propensity score matching analysis, there was no statistically significant difference between the incidences of OIC in patients with and those without prophylactic laxatives (49.3% vs 48.8%, OR = 086, 95% CI: 0.79–1.32). After propensity score matching analysis, the incidence of OIC in patients with prophylactic laxatives was not significantly different from that in patients without prophylactic laxatives (47.8% vs 47.0%, OR = 0.87, 95% CI: 0.74–1.43).

## Discussion

The propensity score matching analysis showed that the incidence of OIC in patients with and without prophylactic laxatives was comparable, suggesting that prophylactic laxatives are not necessarily required when initiating opioid therapy. In addition, age ≥ 65 years and ECOG PS ≥ 3 were shown in multivariate logistic analysis to be significant risk factors in the incidence of OIC.

We found that age ≥ 65 years was a risk factor for OIC. Previous studies also showed that age ≥ 50 years [20] and increased age [22] were associated with a higher incidence of OIC. Constipation occurs due to structural and functional changes of the anorectum in elderly people [23], so elderly patients may be especially prone to constipation. However, the association between OIC and age varies in the studies: one study found no association with age [24], while another study found that age ≥ 65 years decreased the risk of OIC [17]. Further investigation of the potential relationship between OIC and age is therefore warranted. We also found that ECOG PS ≥ 3 was another risk factor for OIC. To the best of our knowledge, the previous studies did not examine the association between performance status and the incidence of OIC. Poor general performance status has been associated with a higher incidence of constipation in patients with cancer and receiving palliative care [25]. In patients receiving chemotherapy, ECOG PS ≥ 2 has been reported to have an association with higher severity of constipation [26]. This evidence suggests that the patients with decreased physical performance status are prone to constipation.

Our results suggest that prophylactic laxatives do not affect the incidence of OIC. In our study, prophylactic laxatives were defined as regularly administered laxatives initiated on day 0 according to the OIC-J study [16]. Furthermore, Higashibata et al. regarded early medication as initiating laxatives within 3 days after initiating opioid therapy [21]. We redefined prophylactic laxatives as initiated regularly on day 0, 1, or 2, and we performed sensitivity analysis. Similar results showing that prophylactic laxatives do not affect the incidence of OIC were obtained.

Despite using the same OIC criteria as the J-RIGID study, our findings were different: the J-RIGID study found significant effectiveness of prophylactic laxatives in preventing OIC. The incidence of OIC with prophylactic laxatives in our study (48.1%) was higher than that in the J-RIGID study (33.7%), although the incidences were comparable between our study and the J-RIGID study in those without prophylactic laxatives (48.9% vs 54.6% respectively) [5]. Comparing patient background, performance status was different between these studies. While the J-RIGID study did not include ECOG PS ≥ 3 patients [5], our study included 305 patients (32.9%) with ECOG PS ≥ 3. Our study showed that ECOG PS ≥ 3 is a risk factor for OIC, so the difference in performance status might have affected the results. In actual clinical settings, performance status of patients receiving strong opioid analgesics vary, from ECOG PS 1 to 4. Therefore, our result is suggested to reflect the real-world clinical practice.

Various types of laxatives are currently available for clinical use. In our study, osmotic laxatives, stimulant laxatives, PAMORA, other types, either alone or in combination, were administered, with osmotic laxatives being utilized in approximately 50% of patients. Naldemedine has become part of clinical practice and is recommended for patients with OIC refractory to traditional laxatives [11, 12]. Several studies have demonstrated the effectiveness of PAMORA in treating OIC [27, 28]. Furthermore, Hamano et al. recently reported that naldemedine prevented constipation and improved constipation-related QOL in patients with cancer in whom opioid therapy was initiated [15]. Ozaki et al. compared prophylactic naldemedine with prophylactic magnesium oxide on the incidence of OIC. The incidence of OIC was found to be significantly lower in patients receiving naldemedine compared with those receiving prophylactic magnesium oxide [29]. In our study, PAMORA was used as a prophylactic laxative in 24.6% of patients. Although the incidence of OIC in patients with prophylactic naldemedine (36.4%) tended to be lower than in those receiving other treatments (osmotic laxatives 54.9%, stimulant laxatives 43.6%, others 57.1%, and their combinations 47.8%), no statistical difference was found. We examined the incidences of OIC with different types of laxatives and found that the incidence of OIC tended to be lower with naldemedine. Based on these results, further study is needed to determine whether naldemedine should be used as the first-line therapy for preventing OIC.

Several limitations of our study need to be considered. First, it may be difficult to generalize the findings as our study was performed in a single center. Second, the retrospective nature of our study may reduce the validity of the data. Although propensity score matching analysis was used to adjust for comparative bias in patient background as much as possible to enhance the level of evidence, an RCT is needed to investigate the more accurate effects of prophylactic laxatives. Third, all eligible patients in our study were inpatients. Hospitalization was shown to be one of the risk factors contributing to constipation in patients receiving palliative care [30]. Therefore, our results may not apply to outpatients. Fourth, some studies have reported that transdermal fentanyl and buprenorphine have significantly lower incidence of OIC than slow-release oral morphine [31, 32]. Therefore, the type of opioid analgesics used may affect the preventive effect of prophylactic laxatives on OIC. Some patients in our study had a change in the type of opioid analgesics changed during the observation period. Therefore, we did not perform a subgroup analysis based on the type of opioid analgesics. Future studies are needed to determine whether the incidence of OIC with prophylactic laxatives varies by the type of opioid analgesics used. Finally, our data were collected from medical records over an 11-year period, during which several types of laxatives, especially naldemedine, were used.

## Conclusions

We retrospectively examined the effect of prophylactic laxatives on OIC using propensity score analysis. Prophylactic laxatives did not affect the incidence of OIC in our study. Our results suggest that prophylactic laxatives are not necessarily required when initiating opioid therapy. In addition, we found that age ≥ 65 years and ECOG PS ≥ 3 were associated with a higher incidence of OIC.

## Supplementary Information

Below is the link to the electronic supplementary material.

Supplementary file1 (DOCX 22 KB)

## Data Availability

No datasets were generated or analysed during the current study.
