# Supplementary material for: Efficacy of prophylactic laxatives against opioid-induced constipation: retrospective propensity score matching analysis
Source: Support Care Cancer. 2025 Jan 21;33(2):115. doi: 10.1007/s00520-025-09154-w (PMC11750890; doi:10.1007/s00520-025-09154-w)
Supplement: Supplementary file 1 — Supplementary file1 (DOCX 22 KB) [file 520_2025_9154_MOESM1_ESM.docx]

**Supplementary Table 1.** Characteristics of the patients after propensity score matching analysis

|  |  | **With prophylactic laxatives**  **(n = 268)** | **Without prophylactic laxatives (n = 268)** | **P value** |
| --- | --- | --- | --- | --- |
| Age (years) | Median (range)  ≥65 (%)  <65 (%) | 68 (29-92)  157 (58.6)  111 (41.4) | 67 (22-93)  150 (56.0)  118 (44.0) | 0.389 |
|  |  |  |  | 0.541 |
| Sex | Male (%)  Female (%) | 177 (66.0)  91 (34.0) | 177 (66.0)  91 (34.0) | 1.000 |
| BMI (kg/m2) | Median (range)  ≥21 (%)  <21 (%) | 21.2 (13.1-37.1)  140 (52.2)  128 (47.8) | 21.1 (11.7-36.6)  137 (51.1)  131 (48.9) | 0.569 |
|  |  |  |  | 0.795 |
| Type of cancer | Gastrointestinal (%)  Others (%) | 22 (8.2)  246 (91.8) | 24 (9.0)  244 (91.0) | 0.758 |
| ECOG PS | ≤2 (%)  ≥3 (%) | 192 (71.6)  76 (28.4) | 184 (68.7)  84 (31.3) | 0.450 |
| Timing of anti-cancer treatment | Anticancer treatment (%)  Best Supportive care (%) | 192 (71.6)  76 (28.4) | 179 (66.8)  89 (33.2) | 0.224 |
| Presence of cancerous peritonitis | Yes (%)  No (%) | 38 (14.2)  230 (85.8) | 44 (16.4)  224 (83.6) | 0.471 |
| Previous gynecological or abdominal surgery | Yes (%)  No (%) | 74 (27.6)  194 (72.4) | 73 (27.2)  195 (72.8) | 0.923 |
| Presence of diabetes | Yes (%)  No (%) | 52 (19.4)  216 (80.6) | 50 (18.7)  218 (81.3) | 0.826 |
| Use of benzodiazepine | Yes (%)  No (%) | 71 (26.5)  197 (73.5) | 63 (23.5)  205 (76.5) | 0.425 |
| Types of opioid analgesics | Morphine (%)  Oxycodone (%)  Fentanyl (%)  Hydromorphone (%)  Tapentadol (%) | 15 (5.6)  198 (73.9)  35 (13.1)  30 (11.2)  11 (4.1) | 9 (3.4)  210 (78.4)  29 (10.8)  25 (9.3)  8 (3.0) | 0.208  0.224  0.424  0.476  0.483 |
| Administration route of opioid analgesics | Oral (%)  Not oral (%) | 239 (89.2)  29 (10.8) | 239 (89.2)  29 (10.8) | 1.000 |
| Daily dose of opioid analgesics  (morphine equivalent) (mg/day) | Median (range) | 30 (9.6-120) | 30 (10-204) | 0.293 |
| Use of weak opioid analgesics | Yes (%)  No (%) | 97 (36.2)  171 (63.8) | 102 (38.1)  166 (61.9) | 0.655 |
| Use of laxatives before initiating strong opioid analgesics | Yes (%)  No (%) | 87 (32.5)  181 (67.5) | 80 (29.8)  188 (70.2) | 0.514 |
| Types of prophylactic laxatives | Osmotic laxatives (%)  Stimulant Laxatives (%)  PAMORA (%)  Others (%)  Combination therapy (%) | 133 (49.6)  39 (14.6)  66 (24.6)  7 (2.6)  23 (8.6) | NA | NA |

BMI, body mass index; ECOG PS, Eastern Cooperative Oncology Group Performance Status; BSC, Best Supportive Care; PAMORA, peripherally-acting μ opioid receptor antagonist; NA, not applicable
